# Patient-Centered Design of an Information Management Module for a Personally Controlled Health Record

**DOI:** 10.2196/jmir.1269

**Published:** 2010-08-30

**Authors:** Colin M Sox, William M Gribbons, Beth A Loring, Kenneth D Mandl, Rosanna Batista, Stephen C Porter

**Affiliations:** ^4^Children's Informatics ProgramChildren's Hospital BostonBostonUnited States; ^3^Division of Emergency MedicineChildren's Hospital BostonBostonUnited States; ^2^Department of Information Design and Corporate CommunicationBentley CollegeWalthamUnited States; ^1^Department of PediatricsBoston Medical Centre & Boston University School of MedicineBostonUnited States

**Keywords:** Attention deficit disorder with hyperactivity, patient-centered care, personal health record, computerized medical record systems, user-computer interface, comprehension, questionnaires, software design

## Abstract

**Background:**

The development of health information technologies should be informed by iterative experiments in which qualitative and quantitative methodologies provide a deeper understanding of the abilities, needs, and goals of the target audience for a personal health application.

**Objective:**

Our objective was to create an interface for parents of children with attention-deficit hyperactivity/disorder (ADHD) to enter disease-specific information to facilitate data entry with minimal task burden.

**Methods:**

We developed an ADHD-specific personal health application to support data entry into a personally controlled health record (PCHR) using a three-step, iterative process: (1) a needs analysis by conducting focus groups with parents of children with ADHD and an heuristic evaluation of a prerelease version of a PCHR, (2) usability testing of an initial prototype personal health application following a “think aloud” protocol, (3) performance testing of a revised prototype, and (4) finalizing the design and functionality of the ADHD personal health application. Study populations for the three studies (focus groups and  two usability testing studies) were recruited from organizations in the greater Boston area. Study eligibility included being an English- or Spanish-speaking parent who was the primary caretaker of a school-age child with ADHD. We determined subjects’ health literacy using the Test of Functional Health Literacy in Adults (TOFHLA). We assessed subjects’ task burden using the National Aeronautics and Space Administration **(**NASA) Task Load Index. To assess the impact of factors associated with the time spent entering data, we calculated Pearson correlation coefficients (*r*) between time on task and both task burden and subject characteristics. We conducted *t* tests to determine if time on task was associated with successful task completion.

**Results:**

The focus groups included three cohorts: 4 Spanish-speaking parents with diverse health literacy, 4 English-speaking parents with lower health literacy, and 7 English-speaking parents with higher health literacy. Both the initial usability testing cohort (n = 10) and the performance-testing cohort (n = 7) included parents of diverse health literacy and ethnicity. In performance testing, the prototype PCHRs captured patient-specific data with a mean time on task of 11.9 minutes (SD 6.5). Task burden experienced during data entry was not associated with successful task completion (*P* = .92). Subjects’ past computer experience was highly correlated with time on task (*r* = .86, *P* = .01), but not with task burden (*r* = .18, *P* = .69). The ADHD personal health application was finalized in response to these results by (1) simplifying the visual environment, (2) including items to support users limited by health literacy or technology experience, and (3) populating the application’s welcome screen with pictures of culturally diverse families to establish a personal family-oriented look and feel.

**Conclusions:**

Our patient-centered design process produced a usable ADHD-specific personal health application that minimizes the burden of data entry.

## Introduction

Pediatric chronic disease management requires timely and effective information exchange between health providers and parents of affected children. Parents’ reporting of health data is the first and most elemental information task in a series of collaborative steps between a parent and a pediatric health provider that result in health-promoting actions. Attention-deficit/hyperactivity disorder (ADHD) is an archetypal pediatric chronic disease where parents’ ability to communicate information to health providers directly impacts management [1,2]. Optimal disease control requires medication dose titration to optimize behavior control with mitigation of side effects—a process that is difficult for practices and populations to make operational [3].

While the classic model of office-based and paper-driven information exchange with the physician as the locus of control often fails to gather data needed for ADHD care [3], electronic health information technologies offer innovative alternatives. A personally controlled health record (PCHR) [4,5] provides an electronic mechanism for patient-driven data capture and communication with health providers [6,7]. Although such PCHRs are endorsed for their potential to impact chronic disease management [8,9], examples of the functionality and effectiveness of products are limited. The user interface of a well-designed personal health application should provide a mechanism for the collection and organization of information from patients with a range of health literacy and technology skills, while maintaining the capacity to codify answers and communicate them in a standardized fashion to clinician-controlled electronic medical record systems.

Successful implementation of a technology depends on a match between the system design and the users’ expectations and abilities. It has long been recognized that employing a user-centered design and development process is essential for ensuring a quality user experience [10-12]. Technology quality has been defined by the ease of learning, overall workload requirements of task completion, error avoidance and reduction, and meeting predetermined performance metrics [11,12]. The degree to which a given system maps to the knowledge and ability of the user determines the quality of that technology experience. By employing a user-centered development process, the likelihood of achieving a successful technology-to-user match is increased significantly.

In the current study, we limited our design focus to the tasks surrounding parents’ one time data entry of a child’s current ADHD status, assuming that any long-term success of a patient-driven technology requires a successful first experience for parents, as has been show in usability research in e-commerce [13,14]. The focus of our formative design process and iterative usability testing was on parents’ needs, expectations, and performance [15,16]. The goals for development were (1) to create a Web-based interface for parents of children with ADHD to enter disease-specific information, (2) to implement a universal design with a visual layout, navigational cues, and workflow that is usable by parents across a range of health literacy and technology skills, and (3) to create an interface to facilitate data entry with minimal task burden.

## Methods

### Prototype Development

Over many decades, a technology development process has emerged to address the needs of the user. The first stage in this process is a needs assessment in which users’ goals and expectations of the task are determined, typically through observations, interviews, and focus groups [10,17-19] before being articulated as specific user requirements. Next, various design prototypes are explored to identify the optimum implementation of a given requirement. This is accomplished through a series of prototypes and participatory walk-throughs with actual or intended users [11,17]. Finally, the implemented design is tested to assess the degree of match with the users' mental models, error prevention and recovery, and overall learning and workload requirements [12,20]. Employing a user-centered development strategy significantly increases the likelihood of a successful technology implementation.

To achieve the first step in information management to inform ADHD care—getting accurate data from parental reporters via an interface that imposed minimal task burden—we followed an iterative three-step development process. First, we concurrently performed a user-centered needs analysis using focus groups and a heuristic evaluation [21] of the main menu and medication data entry pages of a prerelease version of a PCHR before any ADHD-specific application had been created. During this phase, a three-person panel composed of a clinical informatician, usability specialist, and software engineer developed the requirements used to create the application. The discussions and final consensus for the panel’s decisions were informed by the formative data gathered through heuristic review and focus groups. From this work, a preliminary prototype was developed, which we then tested following a “think aloud” protocol with parents of diverse health literacy and technology skills to explore how parents experienced data entry tasks. Redesign based on this usability experiment produced a second prototype with which we completed performance tests with a different cohort of parents focused on time on task, the task experience (as measured by the National Aeronautical and Space Administration [NASA] Task Load Index), and parents’ success in completing specific data entry tasks. Results from the performance test were used to finalize the design of the ADHD data entry tool.

### Study Population

Three subcohorts of subjects for the three studies detailed below (ie, focus groups, initial usability testing, and performance testing) were recruited by a research associate from a broad coalition of organizations in the greater Boston area, including the Children’s Hospital Neighborhood Partnership and the Pediatric Practice Organization at Children’s. Study eligibility required being an English- or Spanish-speaking parent or guardian of a school-age child (5 to 18 years of age) reported to carry a diagnosis of hyperactivity, impulsivity, or attention-deficit/hyperactivity disorder (ADHD). Parents had to have confirmed they were the child’s primary caretaker responsible for communicating with the child’s primary care physician and school. For the usability testing, these inclusion criteria were further restricted to require the index child to have been currently taking a stimulant medication for at least 2 months.

Subjects’ health literacy level was determined based on parents’ completion of the brief Test of Functional Health Literacy in Adults (TOFHLA) [22,23]. We defined lower health literacy as TOFHLA scores of 80 or less. We assumed that subjects would be (1) knowledgeable about the child’s medical history and recent behavior and (2) motivated reporters.

### Baseline Needs Analysis

#### Needs Assessment: Parents as Information Providers

The following 3 focus groups of parents were assembled from the study population (see above): (1) four English-speaking parents with lower health literacy and/or lower educational achievement (high school education or less), (2) seven English-speaking parents with at least a college education and higher health literacy, and (3) four Spanish-speaking parents with diverse health literacy. Experienced moderators (authors BL and RB) followed focus group scripts to encourage discussion of (1) words used in ADHD care, (2) medications, (3) forms that parents are asked to fill out, (4) use of computers to input health data, and (5) parents’ preferences and ideas for how behavioral data and medication data might be displayed using preliminary design sketches as examples. Our process was consistent with the methods espoused by Gibbs [24].

For analysis of the focus group data, we employed a variation on content analyses [20] commonly used in the social sciences, such as grounded theory [25] and narrative explanatory model [26] methodologies. We first assembled and reviewed the collected data (ie, audio recordings, notes, and transcripts) generated for each focus group. Data for each group session were examined to identify majority opinions, dissenting opinions, examples, and anecdotes from the 5 topic areas that framed the discussion for that particular group. Summative opinions and recurring terms were then compared across the 3 focus groups to identify any difference in the pattern of collected data. A group of 3 individuals (the 2 moderators and the observer for the sessions) discussed the data and arrived at consensus for what findings represented a uniform result or a difference based on language or health literacy. The consensus findings were then separately reviewed by the first author to confirm their validity against the interview transcripts. No formal reliability measures were employed during the analysis of this qualitative data.

We offer the following demonstration of how the categorization of the focus group results informed the development of the initial prototype. Asked to explain the term “impulsive,” parents uniformly gave a consistent description. In contrast, presented the term “inattentive,” parents with lower health literacy stated “argumentative” and “provocative” were similar to “inattentive,” while parents with higher health literacy stated that “can’t focus” was similar to “inattentive.” This discrepancy in the interpretation of a core ADHD symptom reinforced the need for the interface to use language familiar to all users.

#### Heuristic Evaluation

Specific displays for the user-interface (the viewer) of a prerelease version of the Indivo platform were analyzed by a single physician-informatician trained in human factors and information design. The main menu page and the medication data entry page were examined from the perspective of a parent who would engage the system for the first time with the intent of initiating documentation/tracking/management for ADHD. The informatician examined each display for its information density, structure, semantics, and navigational cues with the intent of discovering opportunities to improve how the system would support culturally diverse parents with a range of skills in the areas of technology and health literacy.

#### Initial Prototype: Design and Architecture

In response to the finding of the needs assessment and heuristic evaluation, detailed in the “Results” section, the prototype was developed within a prerelease version of Indivo, an open-source PCHR with strong security features and robust interoperability for data exchange between electronic health data sources [4,15]. To create the capacity for childhood ADHD clinical information management within Indivo, we initiated the development of a personal health application to support patient-driven data entry as a critical first step in designing an information management system that would demonstrate value longitudinally.

**Figure 1:**
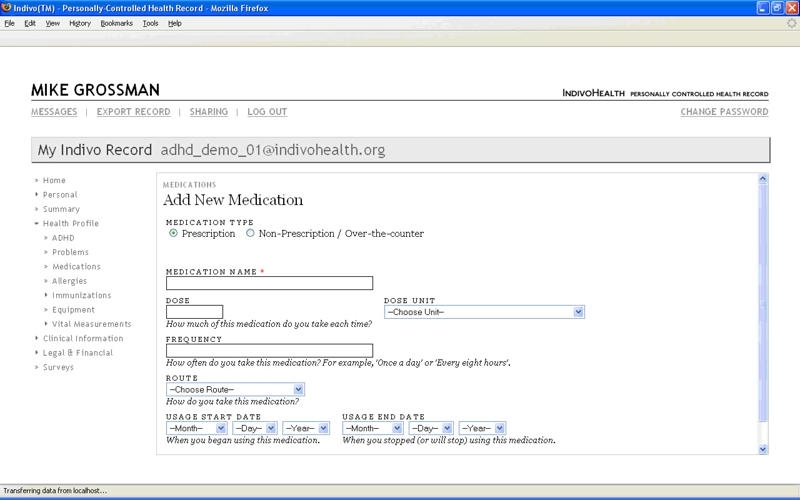
Screenshot from medication entry screen of the prerelease Indivo user-interface used in the heuristic analysis

### Initial Usability Testing

#### Cognitive Walk-through Protocol 

The initial prototype was tested in July 2007 at the Bentley Design and Usability Center (Bentley University, Waltham, MA, USA) using a cognitive walk-through protocol in which subjects were “instructed to verbalize their thoughts as they perform a particular experimental task” [27]. A trained facilitator led each subject through the protocol using a standard script that invited participants to enter data about their child’s ADHD medications and behaviors beginning with the phrase, “I will ask you to perform several tasks using the computer to enter information about your child.” (The full script of the test is available in Multimedia Appendix 1). During testing, we collected observations made about usability issues such as how subjects chose to enter data (eg, typed medication names as free text or selected from a drop-down list). While using the initial prototype, subjects were invited to describe their reactions to it [20,27]. For example, subjects entering data might ask themselves, “Am I receiving adequate confirmation of my actions?” The facilitator further explored subjects’ emotional responses and opinions generated by entering data. To expose problems that would otherwise not be captured, subjects were asked to explain comment or intents of initiated actions. Collected data included audiovisual recording of users’ verbal reactions and facial expressions, and on-screen mouse movements. Each testing session was conducted individually and lasted approximately 1.5 hours.

Using well-accepted social science analytic methods [28], we analyzed and summarized the collected data (ie, video recordings, notes, and NASA index ratings) to identify the usability issues and their causes, which included areas of confusion, unfamiliar or ambiguous terminology, lack of consistency across screens, navigation issues, and the like. After the multidisciplinary team analyzed the rich qualitative data generated by following the think-aloud protocol during the initial usability tests, the team prioritized the findings before generating recommendations for revising the personal health application. Subjects’ computer experience was determined by subjects’ reports and observations made by investigators during testing sessions [22,23], which was then categorized as high, moderate, or low (ie, does not use computers).

#### Initial Prototype Redesign

**Figure 2:**
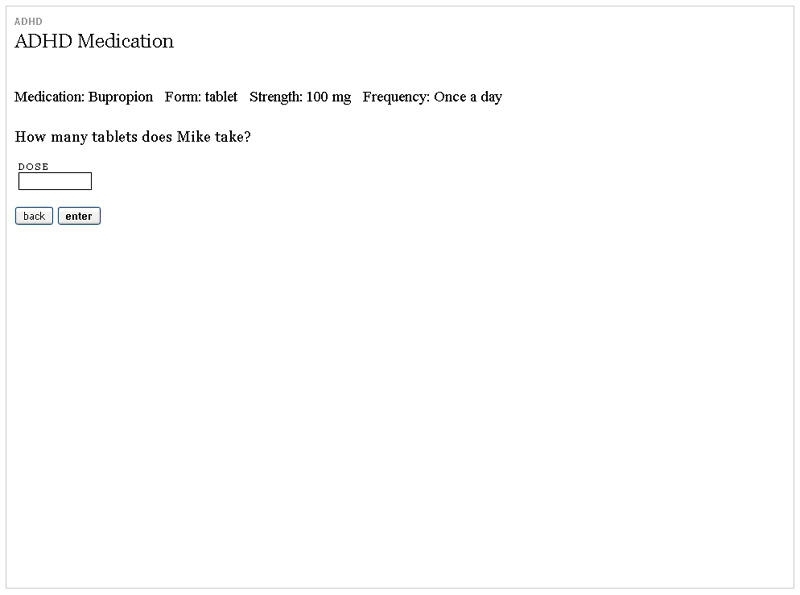
Screenshot from the medication module of the revised prototype used in the performance testing of the ADHD personal health application

In response to comments from initial user testing (see “Results” section and Table 3, which summarizes the changes made to the initial prototype in response to the identified problems), the prototype was refined before it underwent performance testing. Of note, the medication module was significantly redesigned with specific attention to semantics around dose and frequency (see Figure 2).

### Performance-Based Usability Tests Using the Second Prototype

We conducted a series of performance tests of the redesigned prototype with 7 subjects, none of whom had been involved in the initial think-aloud testing. Subjects in the performance tests had a range of health literacy, education, and computer experience (see Table 1 for subject characteristics).

During performance tests, the facilitator introduced the general task to the parent following a script that introduced the performance test to parents reminding them of the general purpose of the reporting tool and that they would complete the task independent of help from the observer in the room (see Multimedia Appendix 2). To minimize contamination of performance data, facilitators did not intervene or assist subjects in task completion. Subjects were observed until they said they had completed all task steps or had reached task failure and had no desire to continue. Performance testing lasted 1.5 hours during which audio-visual recording similar to the think-aloud test was maintained. The primary outcomes assessed included successful task completion, the task completion time, and the NASA Task Load Index, an established measure of task burden [29].

As participants in performance testing did not think aloud (unlike the initial usability tests), analyses relied primarily on task success and failure data as well as observed areas of confusion. To determine if time on task was linearly associated with NASA Task Load Index scores or subject characteristics (ie, previous computer experience), we calculated Pearson product-moment correlation coefficients. We conducted *t* tests to determine if time on task was associated with successful task completion. 

## Results

### Baseline Needs Analysis

#### Needs Assessment

We found that parents attached a range of descriptors to ADHD as a condition. Some descriptors were specific to the formal medical definition (eg, hyper, disorganized, and hard to control), while others addressed comorbid conditions (eg, anxious, angry, and fixation on certain topics).

Parents were shown alternative presentation formats for a behavioral questionnaire with 55 items. Formats displayed included one question per screen, groups of questions, and a longer list that would require scrolling. This questionnaire included the previously validated Vanderbilt Parent Assessment Scale, [30] which collects data on ADHD behaviors necessary for medication-related management decisions. Overall, participants preferred questions presented as one long list, and cited the efficiency of that approach. Of note, only one parent in the lower education, lower health literacy group preferred the one-question-per-screen approach.

Parents were shown two approaches for selection of a medication name: (1) a text box that captured spelling and sounded features of entered letters that would match to a medication name, and (2) a vertically-oriented alphabetical list of all prescription ADHD medications with an index of horizontal letters and a scroll bar to facilitate rapid localization of a given medication name. Parents endorsed the list for selecting medications, recognizing that the names were already spelled-out correctly. Parents also recognized the value of the dynamic text box that could react to the likely name being entered.

#### Heuristic Evaluation

The information-sharing goal for the parent was to provide (or confirm) enough information on behaviors, current medication use, and potential side effects to allow for inferences regarding disease control and potential management options. The main menu display emphasized storage and retrieval of information at the summative level but did not show a clear roadmap for data entry. Users challenged by health literacy or technology experience would ask, “Where do I start?” and “What can I do with each of these labels in the index?”

The view of the main menu of the prerelease Indivo displayed the label “medication” in the left index; selecting the “add new medication” function led to a single screen with 7 data elements (either text box or drop-down menu) awaiting data entry (Figure 1). The accuracy of the data entered into the text box depended on the users’ keyboard-specific skills, medical knowledge, and comprehension of the labels. For example, prior research with parents identified the concept of “dose” as problematic and subject to multiple interpretations [31].

#### Needs Analysis

**Figure 3:**
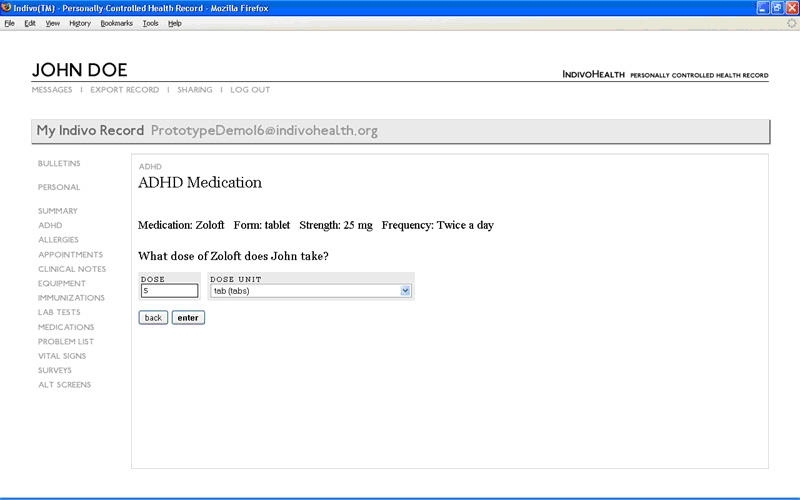
Screenshot from the medication module of the initial prototype of the ADHD personal health application tested following a think aloud protocol

The focus groups and heuristic evaluation each confirmed the need for a disease-specific personal health application that provides a tailored data entry environment for the parent of a child with ADHD. Table 1 summarizes the main design features of the initial prototype proposed for an ADHD personal health application based on issues identified during the needs analysis. Given the importance of accurate capture of dose-specific information on ADHD medications, a structured and hierarchical approach to entry of data in this domain was designed in which data entry began with name and then moved to form, then strength, then frequency/dose, and lastly to review and edit (see Figure 3 for dose screen). Successful navigation and data entry required users to recognize and interact with drop-down menus and scroll bars using a mouse and/or keyboard.

**Table 1:** Summary of baseline needs analyses and heuristic evaluation linked to design of initial prototype of the ADHD personal health application

Identified Problem or Challenge	Findings of Needs Analysis and Heuristic Evaluation	Design Features Embedded Within Initial Prototype
How will the interface support an action-oriented task experience?	The interface will need to provide location and direction to the task experience.^a^ The interface will need to match parents’ goals for the ADHD personal health application.^a^	Immediately display a main menu page that summarizes tasks. Provide an “introduction” step that explains the tasks that follow. Number the steps in order.
How can the interface best support data entry for medications?	Choosing from a list reduces concern about spelling.^b^ A drop-down menu approach to details specific to form and strength will require a stepwise process of data entry.^a^ Limit medical jargon during data entry process^a^	At medication name entry, offer choice of using either dynamic text box or alphabetical list. Provide stepwise data entry for each attribute of a medication. Use of bread crumb display on-screen of previously selected attributes for a medication supports stepwise data entry.
How can a survey on behavior be displayed while simultaneously supporting users’ understanding and limiting missing data?	Having all questions on one screen makes it easier to review answers from previous items.^b^ A limited number of questions per screen facilitates reading. ^b^	Display all survey items on one screen with scroll bar for navigation. Embed an audio file for each question in the survey to augment comprehension.

^a^ Finding from the heuristic evaluation

^b^ Result of focus group sessions

### Cognitive Walk-through Usability Testing Results

The 10 participants in the cognitive walk-through testing had TOFHLA scores ranging from 79 to 100 (see Table 2). Of the 2 subjects with TOFHLA scores less than or equal to 81 and low computer experience, 1 had not graduated from high school (see Table 2) and neither had used the Internet to find health-related information. 

**Table 2:** Characteristics of subjects in both usability testing sessions

Subject ID		Education	Race/Ethnicity^a^	Health Literacy (TOFHLA Score)	Computer Experience
**Cognitive walk-through testing**					
	CW-1	College, some	Black	98	Moderate
	CW-2	College graduate	Mixed	94	Moderate
	CW-3	High school, some	Black	81	Low
	CW-4	Post-college degree	White	100	High
	CW-5	College, some	Black	100	High
	CW-6	High school graduate	Mixed	98	Moderate
	CW-7	College, some	Black	100	High
	CW-8	High school graduate	White	100	High
	CW-9	High school graduate	White	98	High
	CW-10	High school graduate	Mixed	79	Low
**Performance testing**					
	PT-1	High school graduate	White	98	Moderate
	PT-2	College graduate	White	100	High
	PT-3	Graduate school degree	White	98	Moderate
	PT-4	College graduate	Black	98	High
	PT-5	College, some	White	96	High
	PT-6	Grade school, some	Black	80	Low
	PT-7	College, some	Mixed	98	High

^a^ Subjects’ race/ethnicity was determined by self-report concordant with NIH policy *NOT-OD-01-053*.

^b^ This assessment is a combination of subjects’ reports of past experience and observations made by examiners during usability testing.

#### Overall Impressions

Subjects’ overall reaction to the concept of the personal health application was positive, as emphasized by CW-1: “I think it’s awesome! It would save time by entering information before the visit; the doctor would be better prepared.” All subjects found the progression of data entry tasks intuitive, and most liked the feedback they received after completing each step. Problems identified during the usability tests are presented in Table 3. While most found the homepage easy to use, two felt it was visually unpleasant (eg, “bland,” and “clinical”). The purpose of the computer system was unclear to some (ie, they did not know whether it was to get or give information). Some subjects felt there should be a help button on the menu.

#### Medication Module

Subjects endorsed the concept of a medication summary page. Notably, 8 of 10 subjects failed the medication data entry task for which success required a parent to enter all attributes of the medicine (ie, name, form, strength, dose, and frequency), review the summarized data on-screen, and endorse it as correct. The attributes of dose and frequency created the most confusion. For example, a subject with higher health literacy and with moderate computer experience (CW-1) remarked: “I don’t understand…what [does] it mean by ‘dose?’” before leaving the field blank. While entering data on dose, a well-educated subject with higher health literacy and high computer experience (CW-5) commented: “Oh, wait a minute…two doses (shakes head)…two tablets; go back (smiles)…one tablet twice a day; there you go (smiles while laughing).” Of note, two parents (CW2 and CW8) entered both name and strength information (ie, Concerta 27 mg) into the dynamic text search box, which caused an error in returned matches and ultimate frustration for subjects.

Participants were divided about whether they preferred the dynamic text box or the alphabetized list. A subject with lower health literacy, little computer experience, and no high school degree (CW-3*)* noted: “You know how some people can’t really spell the names of the medications? I would like to see the list because you would be able to say, ‘yeah, that’s the medicine.’” Terminology about medication strength introduced confusion for users—the drop-down list for combination medicines displayed two numbers that represented the strength of each component and was not intuitive. For example, a well-educated subject with higher health literacy and high computer experience (CW-9) noted: “I am not a pharmacist; how am I supposed to know what 10/2.5 is?”

**Table 3:** Summary of problems identified in usability testing and solutions incorporated into second prototype of the ADHD personal health application

Identified Problem	Severity Description/Reason	Design Features Embedded Within the Second Prototype
Purpose of system unclear	Moderate with regard to satisfaction with user experience	Revised introductory video to better explain system
Visually bland	Minor in short term for testing purposes, more significant for actual use in field	No changes for performance prototype
Confusion with meaning of dose and frequency	Severe in that total daily dose exposure cannot be calculated	Revised semantics in plainer language
Entry of too much detail into dynamic search creates errors	Severe in that medication name is primary branch point for all other details	Limited algorithmic matching to first five alphabetical characters entered
Navigation within behavioral survey problematic	Moderate with regard to increased frustration and potential for missing data	Behavioral survey with simplified layout and introductory video explaining navigational features
Confusion with terminology in behavioral survey	Moderate with regard to potential inaccuracies in responses	Text-specific help feature added

### Performance-Based Usability Testing Results

Table 2 describes the relatively diverse group of 7 subjects who participated in the performance tests. Similar to the initial usability testing, most participants liked using the redesigned prototype. For example, subject PT-2 commented: “It’s very simple and extremely easy to use.”

#### Task Completion

Of the 7 subjects, 5 successfully completed all 3 data entry tasks; subject PT-6 failed all three tasks, and PT-2 failed the behavioral symptom task but successfully completed the other two tasks. An example of the importance of providing users feedback about completing an interaction task, PT-2 stated: “It was just confusing, because it doesn’t say ‘thank you for completing the above three steps; you’re finished.’ You are just sort of wondering, ‘Did I miss a question?’” In addition to having lower health literacy, less than high school education, and low computer experience (see Table 1), PT-6’s poor performance was influenced by poor screen-scrolling skills that resulted in his not viewing all the questions.

#### Time Requirement

The mean of all 7 subjects’ total time on task was 11.9 minutes (SD 6.5 minutes, range 7 to 26 minutes, median 9 minutes), with the subject who failed all 3 tasks (PT-6) having the longest time on task (26 minutes) and the subject who failed 1 task (PT-6) having the shortest time on task (7 minutes). Users’ previous computer experience was highly correlated with their time on task (*r* = .86, *P* = .01). For the 5 subjects who successfully finished all 3 data entry tasks, the mean task completion time (TCT) was 8.8 minutes (SD 1.5, range 7 to 11 minutes, median 9 minutes).

Completing the behavioral module took subjects the most time. After restricting analyses to those who completed each task, the mean TCT for the behavioral task was 5.5 minutes (median 4 minutes, range 2 to 5 minutes) compared with the medication module’s mean completion time of 2.2 minutes (median 3 minutes, range 1 to 5 minutes) and the side-effect module’s mean of .72 minutes (median 1 minute, range 0 to 1 minute).

Time on task was not associated with successfully completing all 3 tasks (*P* = .27), but subjects who successfully completed the behavioral module trended toward having briefer time on task (mean 3.6 minutes) than the 2 who didn’t complete this task (mean 10 minutes, *P* = .07). The amount of time subjects reported having spent on completing the 3 tasks (as measured by the temporal subsection of the NASA Task Load Index) was correlated with the actual time they spent on the 3 tasks (*r* = .74, *P* = .06).

#### Task Burden

The aggregate NASA Task Load Index score was not associated with successfully completing all 3 tasks (*P* = .92), nor were scores on the effort and frustration NASA Task Load Index subscales associated with successfully completing all 3 tasks (*P* = .36 and *P* = .67, respectively) . Similarly, users’ previous computer experience was not correlated with their total NASA Task Load Index score (*r* = .18).

Figure 4 presents the task burden experienced by subjects during data entry. Subjects’ responses confirmed the low physical task burden of using the computer even for the individual who did not master the mechanics of the mouse. Although some subjects experienced high frustration, their scores on the frustration subsection of the NASA Task Load Index were not correlated with time demands of the interface (ie, time on task) (*r* = .19, *P* = .69). The two users with the highest frustration scores were college-educated and had high health literacy, with one having high and the other moderate computer experience. No clear differences were evident for median task scores between subjects who did or did not successfully complete all 3 tasks.

**Figure:**
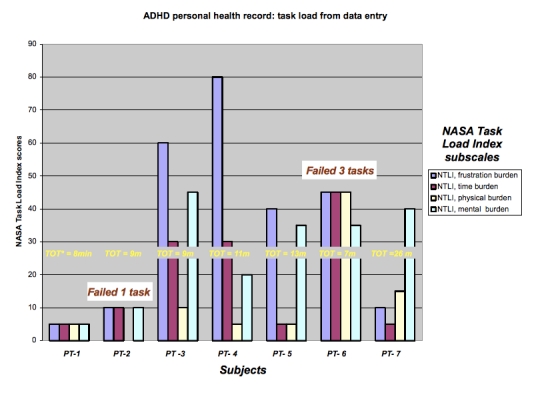
Task burden experienced and time on task (TOT) spent by subjects while they attempting to complete the 3 tasks during the performance testing of the revised prototype of the ADHD personal health application

### Final ADHD Personally Controlled Application Interface

The final version of the ADHD personal health application, which was finalized in response to the performance testing results, simplified and personalized the visual environment (see Multimedia Appendix 3). A panel of pictures showing children and multicultural family units reinforces the pediatric perspective. A line of colored rectangles creates a link across the main menu into the submenu pages. A help button is overtly visible to the user in both the left navigation menu and the top menu on-screen. The main menu presents users an initial message of what task steps are needed as well as confirmation of when a task step has been completed by virtue of a blue circle adjacent to each step changing to a green check mark. To support complete and accurate data entry, a series of solutions are embedded in the final design. In the behavior module, questions skipped by the user in the initial process of answering are redisplayed to encourage completion of each one. To address errors due to specifying dose, semantics around dose-units were further simplified by asking “how many” for solid-based medications and “how much” for liquid-based medications. Dose units are inferred based on the specified form and strength at an earlier point in the hierarchical process of discovery.

The final version of the ADHD personal health application includes items to support a potential user who is limited by health literacy or technology experience. These include brief videos that demonstrate key features (workflow and tasks) and give hints on navigation (eg, scroll bar use). The items in the behavior module and the medication names are presented with links to audio files. In addition, in the behavior module, alternative text language can be activated by moving the mouse over the terms. The skills that a user must employ (and that are consistent with minimal Web navigation) are to (1) find letters on the keyboard and/or use the mouse to direct the on-screen arrow to the desired location and then click on a given selection, (2) recognize the scroll bar as a tool for navigation, and (3) recognize that a drop-down menu will show potential responses for a given item.

## Discussion

By employing a patient-centered design process, we have successfully developed a gateway for the parent of a child with ADHD to populate a PCHR with data to drive symptom monitoring and clinical decision making. Our universal design approach creates a single solution for display and user-interaction with the software endorsing users’ values for colocating, viewing, and updating health information [32]. Our medication module allows for a parent to provide information on prescribed medications across multiple levels of granularity in order to codify the child’s total daily medication exposure and the timing of doses. Consistent with literature [33], some participants with limited computer skills struggled while using the application; our usability testing suggested that successful data entry by users with limited technology experience may require embedding a brief tutorial in navigation and mouse-directed interactions within the final version of the personal health application.

The final design for the ADHD personal health application successfully matches parents’ expectations while entering data essential to evidence-based treatment decisions. While the drop-down menu creates an important opportunity for controlled vocabulary, the choices offered to users must match up to their own knowledge and practices to ensure accurate responses. Consistent with existing literature [34,35], the study populations of both the focus groups and usability testing met our assumptions about parents’ needs and practices (ie, parents are knowledgeable, observant, and motivated reporters about their children). As we hoped, the redesigned personal health application prototype met subjects’ expectations. Synergy between users’ expectations of a technology and their actual experience is relevant to sustained user-engagement with a technology over time.

Usability testing of the ADHD personal health application revealed that parents who were not familiar with the mouse and had not previously “scrolled” on a Web page were at a significant disadvantage. We do not believe this finding compromises the success of our universal design, as the profile of users for an electronic personal health application will necessarily be engaged in a Web browser experience and thus have basic scrolling and point-click skills. Importantly, our final design embeds multiple mechanisms to support health literacy demands, including alternative text explanations as well as audio files that allow for content to be heard as well as read on-screen.

The final design of the ADHD personal health application ensures maximal completeness and accuracy of parent-entered data. The success of a personal health application in promoting the quality of care delivered for ADHD requires that sufficient and correct information is communicated to the system. The ADHD personal health application is currently being tested as part of a clinical trial comparing paper-based data entry to a computer-based mechanism with this application at its core.

We believe our solution addresses a difficult-to-solve tension between the expectations of a highly educated, technologically proficient parent and a parent who possesses limited knowledge or skill in either the health or technology domains. There is danger in a design appearing “too simple” and, therefore, being perceived by some users as less valuable or less valid as medically oriented software. It was interesting to note that users who voiced frustration during testing of the prototype were more often highly educated parents whose expectations for speed, style, or functionality were not met. However, these expectations were not core to the exchange of data so were not judged to impact the overall task burden for the ADHD personal health application.

The ADHD personal health application does not employ channels such as telephony or objects such as scannable forms used by others in the direct capture of medical data from patients [36-38]. Our goal was to tightly couple the Web-based PCHR construct to the input of data while preserving the ability to flexibly display a range of question types and deploy adjunctive features to assist with accuracy in data capture. Existing systems such as telephone-linked communications, shown to engage and capture valid data from a diverse patient population via structured telephone-based communication, currently do not link to a PCHR entity that allows patients to electronically view/update/reuse previously collected data. Although scannable forms provide a familiar “paper-and-pen” approach for users, constraints on efficient display of items, the potential for incomplete data from users, and the inability to support user’s constrained by reading comprehension make these forms a less than ideal solution.

### Limitations

The success of our personal health application development should be considered a preliminary result. Although our usability testing was rigorous, our sample size was small and nonrepresentative. Performance testing in a usability lab cannot avoid some artificiality in the experience, and, therefore, our parents’ report of task burden is a proxy for what might be reported from a data entry experience at home with stress and noise associated with a full and busy household. As our testing examined a one-time data entry experience, we cannot report on usability issues related to longitudinal engagement with a PCHR where iterative data entry is required, as long-term use can be affected by complex factors unrelated to the interface. 

### Conclusions

Informed by a patient-centered formative process that included measurement of the task experience, we successfully developed a usable patient-centered ADHD-specific personal health application with minimal task burden for parents to enter data about their child with ADHD. This work confirmed the value of iterative usability testing for assessment and improvement of eHealth prototypes where the users’ interactive experiences are critical to the product’s success. Any eHealth technologies that intend to provide patient-centered solutions require a design/re-design process that centers on the voice of the patient.

## Multimedia Appendix 1

Usability test script

## Multimedia Appendix 2

Tasks assessed during performance testing

## Multimedia Appendix 3

Screenshots of the final ADHD PCHR interface

## Abbreviations

ADHD: attention-deficit/hyperactivity disorder
PCHR: personally controlled health record
TCT: task completion time
TOFHLA: Test of Functional Health Literacy in Adults
TOT: time on task

## References

[1] American Academy of Pediatrics, Committee on Quality Improvement, Subcommittee on Attention-Deficit/Hyperactivity Disorder (2000). Clinical practice guidelines: diagnosis and evaluation of the child with attention-deficit/hyperactivity disorder. Pediatrics.

[2] American Academy of Pediatrics, Committee on Quality Improvement, Sub-committee on Attention-Deficit/Hyperactivity Disorder (2001). Clinical practice guideline: treatment of the school-aged child with attention-deficit/hyperactivity disorder. Pediatrics.

[3] Guevara James P, Feudtner Chris, Romer Daniel, Power Thomas, Eiraldi Ricardo, Nihtianova Snejana, Rosales Aracely, Ohene-Frempong Janet, Schwarz Donald F (2005). Fragmented care for inner-city minority children with attention-deficit/hyperactivity disorder. Pediatrics.

[4] Mandl Kenneth D, Simons William W, Crawford William C R, Abbett Jonathan M (2007). Indivo: a personally controlled health record for health information exchange and communication. BMC Med Inform Decis Mak.

[5] Mandl Kenneth D, Kohane Isaac S (2008). Tectonic shifts in the health information economy. N Engl J Med.

[6] Kim Matthew I, Johnson Kevin B (2002). Personal health records: evaluation of functionality and utility. J Am Med Inform Assoc.

[7] Sahnders JH (2008). Robert Wood Johnson Foundation, California HealthCare Foundation.

[8] Simons William W, Mandl Kenneth D, Kohane Isaac S (2005). The PING personally controlled electronic medical record system: technical architecture. J Am Med Inform Assoc.

[9] Tang Paul C, Ash Joan S, Bates David W, Overhage J Marc, Sands Daniel Z (2006). Personal health records: definitions, benefits, and strategies for overcoming barriers to adoption. J Am Med Inform Assoc.

[10] Wixon D, Ramey J (1996). Field Methods Casebook for Software Design.

[11] Mayhew D (1999). The Usability Engineering Lifecycle: A Practitioner’s Handbook for Interface Design.

[12] Nielsen J (2000). Designing Web Usability.

[13] Roy M, Dewit O, Aubert B (2001). The Impact of Interface Usability on Trust in Web Retailers. Internet Research.

[14] Johnson Constance M, Johnson Todd R, Zhang Jiajie (2005). A user-centered framework for redesigning health care interfaces. J Biomed Inform.

[15] Halamka John D, Mandl Kenneth D, Tang Paul C (2008). Early experiences with personal health records. J Am Med Inform Assoc.

[16] Rasmussen J, Pejtersen AM, Goodstein LP (1994). Cognitive Systems Engineering.

[17] Hackos JT, Redish JC (1998). User and Task Analysis for Interface Design.

[18] Jonassen DH, Hannum WH, Tessmer M (1989). Handbook of Task Analysis Procedures.

[19] Schraagen JM, Chipman SF, Shalin VL (2000). Cognitive Task Analysis.

[20] Dumas JS, Redish JC (1999). Tabulating and Analyzing Data. A Practical Guide to Usability Testing. Revised edition.

[21] Dumas JS, Redish JC (1999). Getting Experts to Review the Design. A Practical Guide to Usability Testing. Revised edition.

[22] Parker RM, Baker SW, Williams MV, Nurss JR (1995). The Test of Functional Health Literacy in Adults: a new instrument for measuring patient's literacy skills. J Gen Intern Med.

[23] Baker DW, Williams MV, Parker RM, Gazmararian JA, Nurss J (1999). Development of a brief test to measure functional health literacy. Patient Educ Couns.

[24] Gibbs A (1997). Focus groups. Social Research Update.

[25] Glaser BG, Strauss AL (1967). The discovery of grounded theory: strategies for qualitative research.

[26] Kleinman A (1988). The Illness Narratives: Suffering, Healing and the Human Condition.

[27] Patel VL, Kaufman DR (2006). Cognitive science and biomedical informatics. Shortliffe EH, Perreault LE, editors. Medical Informatics: computer applications in health care and biomedicine. 3rd edition.

[28] Babbie E (2001). The Practice of Social Research. 9th edition.

[29] National Aeronautical and Space Administration (1985). NASA Task Load Index (TLX) Version 1.0 User's Guide.

[30] Wolraich Mark L, Lambert Warren, Doffing Melissa A, Bickman Leonard, Simmons Tonya, Worley Kim (2003). Psychometric properties of the Vanderbilt ADHD diagnostic parent rating scale in a referred population. J Pediatr Psychol.

[31] Porter Stephen C, Cai Zhaohui, Gribbons William, Goldmann Donald A, Kohane Isaac S The asthma kiosk: a patient-centered technology for collaborative decision support in the emergency department. J Am Med Inform Assoc.

[32] Weitzman Elissa R, Kaci Liljana, Mandl Kenneth D (2009). Acceptability of a personally controlled health record in a community-based setting: implications for policy and design. J Med Internet Res.

[33] Kim Eung-Hun, Stolyar Anna, Lober William B, Herbaugh Anne L, Shinstrom Sally E, Zierler Brenda K, Soh Cheong B, Kim Yongmin (2009). Challenges to using an electronic personal health record by a low-income elderly population. J Med Internet Res.

[34] Bussing Regina, Mason Dana M, Leon Christina E, Sinha Karabi (2003). Agreement between CASA parent reports and provider records of children's ADHD services. J Behav Health Serv Res.

[35] Wolraich Mark L, Lambert E Warren, Bickman Leonard, Simmons Tonya, Doffing Melissa A, Worley Kim A (2004). Assessing the impact of parent and teacher agreement on diagnosing attention-deficit hyperactivity disorder. J Dev Behav Pediatr.

[36] Anand V, Biondich PG, Liu G, Rosenman MB, Downs SM (2004). Child Health Improvement through Computer Automation: the CHICA system. Medinfo.

[37] Finkelstein J, Friedman RH (2000). The potential role of telecommunications technologies in the management of chronic health conditions. Disease Management & Health Outcomes.

[38] Friedman R H (1998). Automated telephone conversations to assess health behavior and deliver behavioral interventions. J Med Syst.

