# Supplementary material for: Patient-Centered Design of an Information Management Module for a Personally Controlled Health Record
Source: J Med Internet Res. 2010 Aug 30;12(3):e36. doi: 10.2196/jmir.1269 (PMC2956329; doi:10.2196/jmir.1269)
Supplement: Supplementary file 1 [file jmir_v12i3e36_app1.pdf]

Multimedia Appendix 1: Usability Test Script

Participant Briefing

Welcome: Thank you for coming in today. My name is <name>. I will be working with you on ADHD personal health care record today.

Session Details: Let me tell you what to expect today. If you have questions at any time, please ask. There are three parts to our session:

- First, we'll ask you a few background questions about how you currently use computers.
- Next, we'll ask you to perform several tasks using the computer to enter information about your child. Next, I'll ask you about your experience using the computer program and get your suggestions for improvement.
- At the end of the session, I'll have you complete a standard questionnaire on paper regarding your child's behavior.

The session will last about an hour and a half, so we will be done at [*time*]. Is that OK?

Thinking aloud: As I mentioned, I'm going to give you a series of tasks to work on. While you are working, I'd like you to think aloud. In other words, I'd like to you tell me what you are thinking, describe the steps that you are taking, what you are expecting to see, why you are doing what you're doing, and so on.

Positive and negative comments welcome: Your comments are very important to us, so we ask you to give us your open opinions (both positive and negative) of what you see. You won't hurt my feelings because I didn't design the computer program. Feedback from you and other participants will help us make recommendations for improving the program.

Any difficulties you may run into are likely caused by the design of the computer, not you, or your talents or abilities.

Neutral Observer

My role here is as a neutral observer. I will be taking notes, but will be mostly silent while you're working. If you get really stuck, I will give you some guidance, but for the most part I'd like you to try to figure things out on your own, because that helps us to understand where any design problems exist.

Informed Consent: <discussed here>

Initial Questions

Go through these quickly:

1. Have you ever looked up health information online? If so, where?
2. Have you ever looked up information about medicines online? If so, where?
3. What is your opinion of the way that prescription bottles are labeled?

First Impressions of ADHD Indivo Personal Health Record

Show the ADHD Indivo personal health record main menu.

1. What are your initial impressions of this page?
2. Are there any things you like or dislike about it?
3. What do you think these options are on the left? *(Do they know it's a menu?)*
4. Based on what you see here on the right, what types of things could you do with this program?
5. What would do you do next? *(Note comments about the instructions and their reaction to the Introduction.)*
   Went to Intro? Yes No

Tasks assessed in usability tests following a "Talk Aloud" protocol

TASK 1: Use the site to enter information about the medications you give your child to treat ADHD.

Task Start Time: _________
They went to: Type Name Look at List
Notes and observations:
Assistance required? ü ü ü Yes ü ü ü No Where?
Task End Time:
SUCCESS POINT: Participant successfully enters child's medicine

Task 1B: I noticed that you used the [Type a Name / Look a List] to find the medication. I'd like to get your opinions on the other way to find the medication.

Follow-up questions
1a. What did you think about typing in the medicine name?
1b. What did you think about finding the medicine name in a list? Which do you prefer?
1c. What are your thoughts about the whole process of entering the medication information?
1d. Were there parts of the process that could be made clearer or easier to use?
1e. Did you notice the audio icons or not? Why did you use/not use them?
1f. Do you have any other comments?

TASK 2: Now let's say that you give your child Zoloft 20mg a day twice a day. Please show me how you would enter that information. *<Hand the subject the bottle>*

Task Start Time: _________
They went to: *Type Name* *Look at List*
Assistance required? ü ü ü Yes ü ü ü No Where?
Task End Time:
SUCCESS POINT: Participant successfully enters Zoloft information

Follow-up questions
2a. I noticed that you chose the ________ way to locate the medication this time. Do you have any thoughts on that?
2b. What are your thoughts about the process of entering the Zoloft information?
2c. Were there parts of the process that could be made clearer or easier to use?
2d. Do you have any other comments?

TASK 3: Let's say you wanted to use the program to enter information about your child's behavior. How would you do that?

Goals: 1) Are survey questions answered in succession?
2) Is navigation down the page an issue
3) Do parents who skip an item return to it?
4) Are there issues with choice of "I'm not sure" option?
Task Start Time: _________
Notes and observations:
Assistance required? ü ü ü Yes ü ü ü No Where?
Task End Time:
SUCCESS POINT: Participant successfully completes the Vanderbilt.

Follow-up questions
3a. Tell me what you thought about doing this task.
3b. Do you have any comments about the way the questions were shown?

3c. Do you have any comments about the amount of time it took?
3d. Were there parts of the process that could be made clearer or easier to use?
3e. Do you have any other comments?
NOTE: If they struggled, show them the alternative presentations of questions (one at a time or chunked) in the PowerPoint file.
3f. Here's another possible way of showing the questions. What do you think of this?

TASK 4: I'd like to show you an idea for a calendar that can be used to keep track of behaviors, medications, events, etc. to help you manage your child's ADHD. Let's say you wanted to enter information about your child's behavior last Tuesday. How would you do that?

Goals: Gather input for the design of a successful and useful calendar.

Task Start Time: _________

Notes and observations:

Assistance required? ü ü ü Yes ü ü ü No Where?

____

Task End Time:

SUCCESS POINT: Participant is able to enter info into the calendar.

Follow-up question

4a. Tell me what you thought about doing this task.
4b. What did you think about the screen for entering information? Anything missing?
4c. Would you find the free text boxes useful or not?
4d. Would you use the calendar or not? Why? If so, when/how?
4e. What would make the calendar most useful for you?

Post-Session Interview

1. What are your overall impressions of this computer program?
2. On a scale of 1 to 5, where 1 is "extremely difficult" and 5 is "extremely easy", how would you rate your experience using this program for entering medication information about your child with ADHD?
3. On a scale of 1 to 5, where 1 is "extremely difficult" and 5 is "extremely easy", how would you rate your experience using this program for answering behavioral questions on a survey?
4. On a scale of 1 to 5, where 1 is "extremely difficult" and 5 is "extremely easy", how would you rate your experience using this program for entering information on a calendar?

   extremely difficult 1 2 3 4 5 *extremely easy*
5. What did you like **best** about using the program?
6. What did you like **least** about using the program?
7. Are there any features that you didn't see on the site today that you would find useful?
8. What features do you think you would use most often (Medications, Survey, Calendar)?
9. Do you have any final comments or advice for the designers of this program?

Instructions for the Vanderbilt paper survey: I am going to give you a paper form to fill out called the NICHQ Vanderbilt survey. It is two pages with a number of questions for you to answer. It asks you about your child's behaviors.
This type of form might be mailed to a parent with instructions to "fill it out and return it to the office." Parents often have to fill out this form on their own.

I'd like you to look at the form and read the instructions. Then, answer the questions as best you can. Let me know when you are done.

Need to leave 20 minutes at the end for the paper survey.

Start time: _______ End time: _______

Observations: *[Time permitting]* Do you have any comments about how easy or difficult that was to fill out?
