# Supplementary material for: Patient-Centered Design of an Information Management Module for a Personally Controlled Health Record
Source: J Med Internet Res. 2010 Aug 30;12(3):e36. doi: 10.2196/jmir.1269 (PMC2956329; doi:10.2196/jmir.1269)
Supplement: Supplementary file 2 [file jmir_v12i3e36_app2.pdf]

Multimedia Appendix 2: Tasks assessed during performance testing

Participant Briefing

"This screen is the starting point for you to begin work on your own. Please review the menu and finish the tasks it asks you to do. It will guide you as you enter different types of information about your child's ADHD. I will ask you to work on your own. Please let me know when you believe that you have finished the work of entering information."

Tasks

TASK 1: "Use the site to enter information about the medications you give your child to treat ADHD. You can refer to the prescription bottles you brought with you, if necessary."

Data collected:

a) Time on task
b) Use of the introduction video
c) Observations on navigation of application
d) Frequency audio icon was viewed
e) Frequency video icon was viewed
f) Spontaneous verbalizations of frustration or confusion
g) Task success

TASK 2: "Now let's say that you give your child Zoloft 20mg a day twice a day. Please show me how you would enter that information."

Data collected:

a) Time on task
b) Observations on navigation of application
c) Frequency audio icon was viewed
d) Frequency video icon was viewed
e) Spontaneous verbalizations of frustration or confusion
f) Task success

TASK 3: "Let's say you wanted to use the program to enter information about your child's behavior. How would you do that?"

Data collected:

a) Time on task
b) Frequency audio icon was viewed
c) Frequency video icon was viewed
d) Spontaneous verbalizations of frustration or confusion
e) Task success
f) Frequency questions were missed or not answered on first attempt

TASK 4: "Please show me how you would enter information about side effects your child is having with his or her medication."

Data collected:

a) Time on task
b) Frequency audio icon was viewed
c) Frequency video icon was viewed
d) Spontaneous verbalizations of frustration or confusion
e) Task success
f) Frequency questions were missed or not answered on first attempt
