# Supplementary material for: Patient-Centered Design of an Information Management Module for a Personally Controlled Health Record
Source: J Med Internet Res. 2010 Aug 30;12(3):e36. doi: 10.2196/jmir.1269 (PMC2956329; doi:10.2196/jmir.1269)

## Slide 1
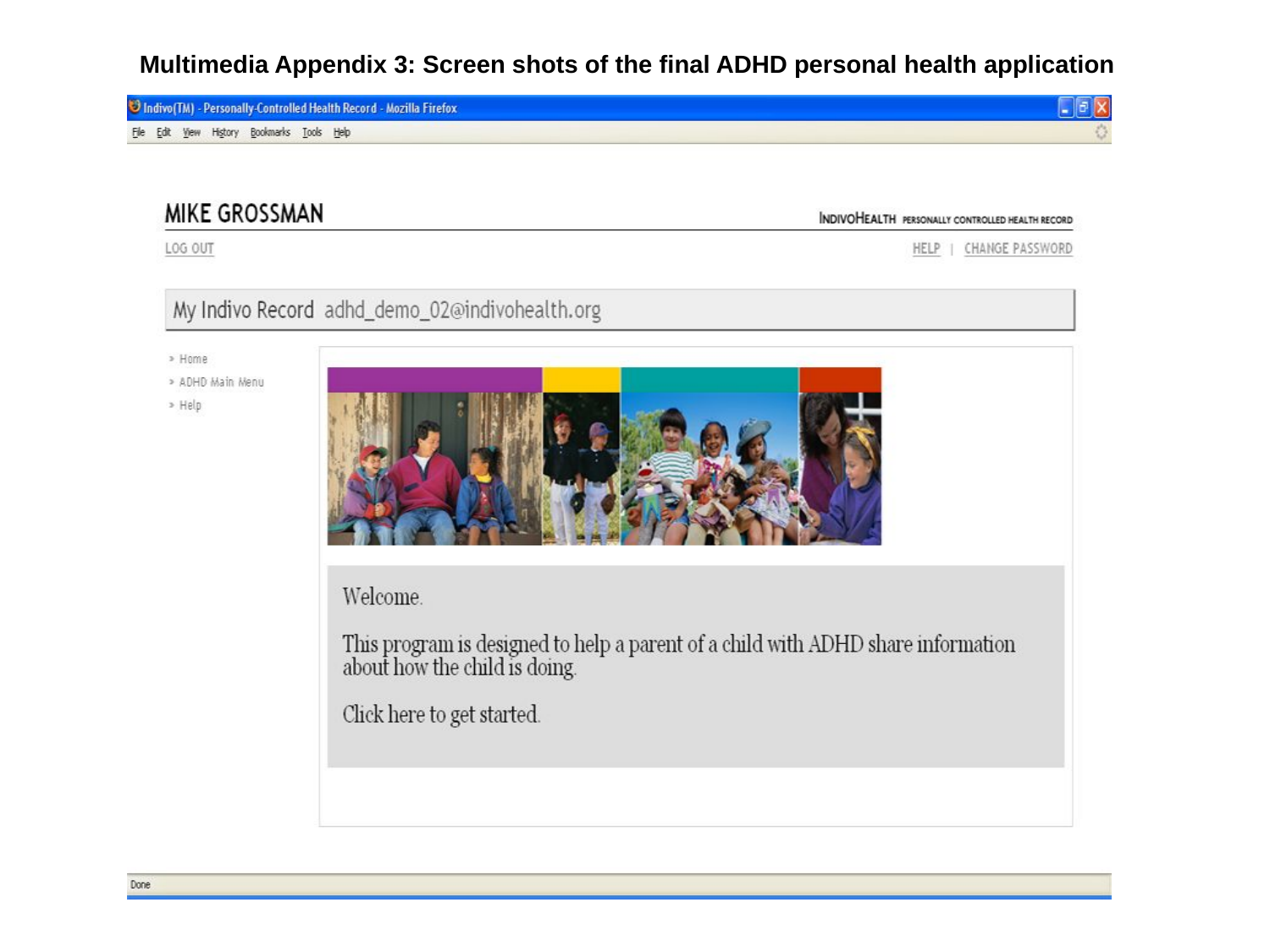

Multimedia Appendix 3: Screen shots of the final ADHD personal health application

## Slide 2
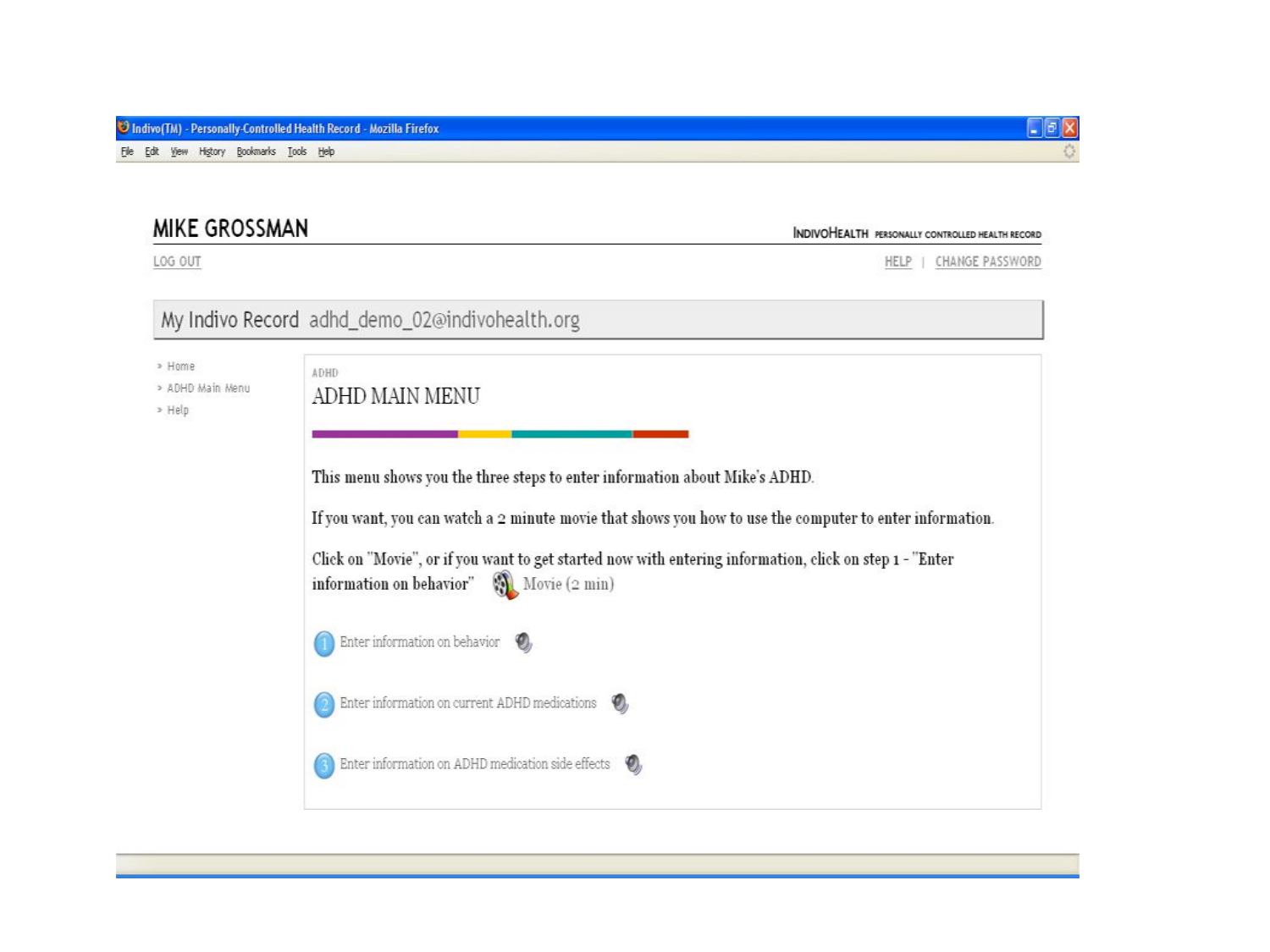

## Slide 3
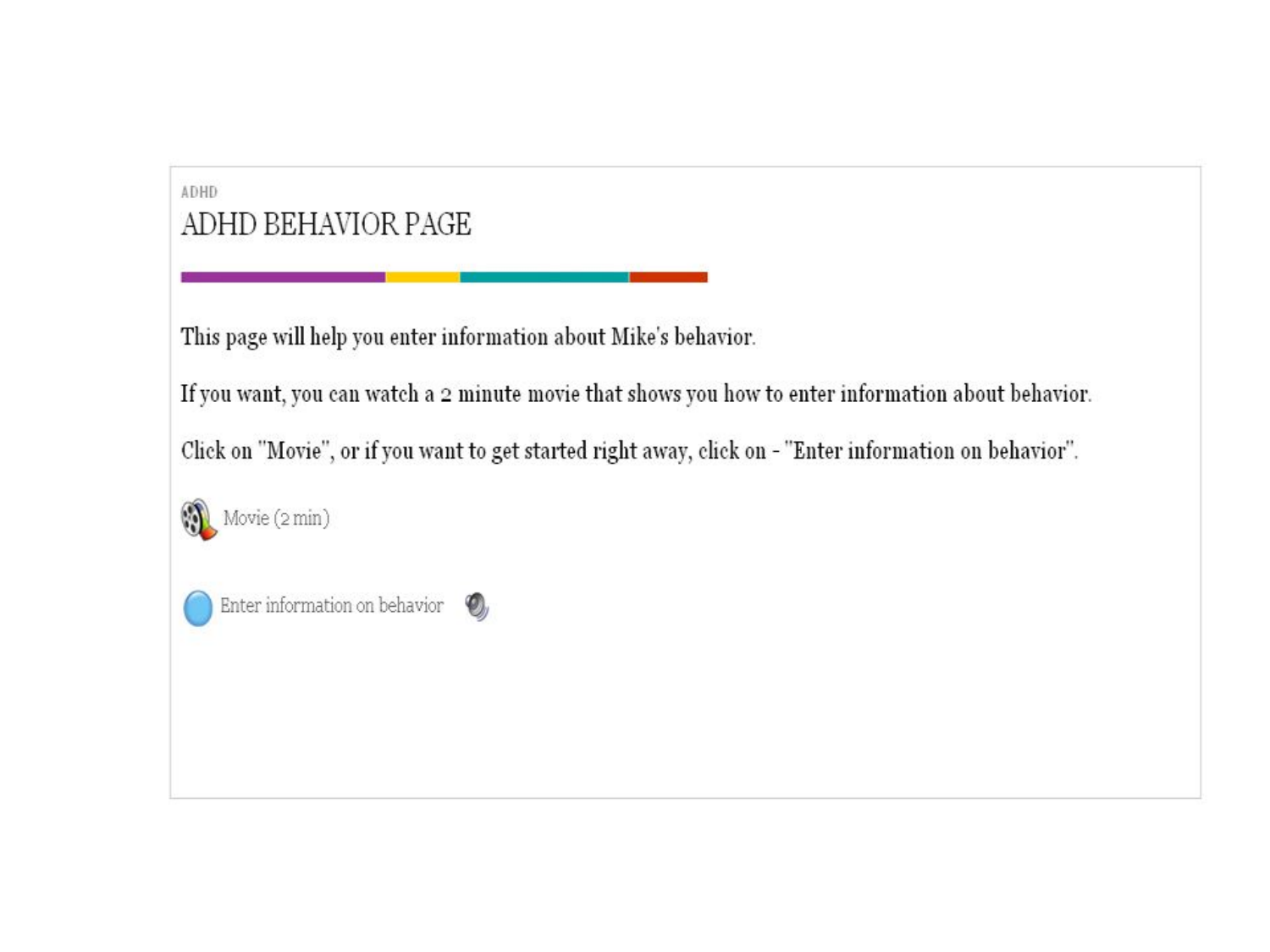

## Slide 4
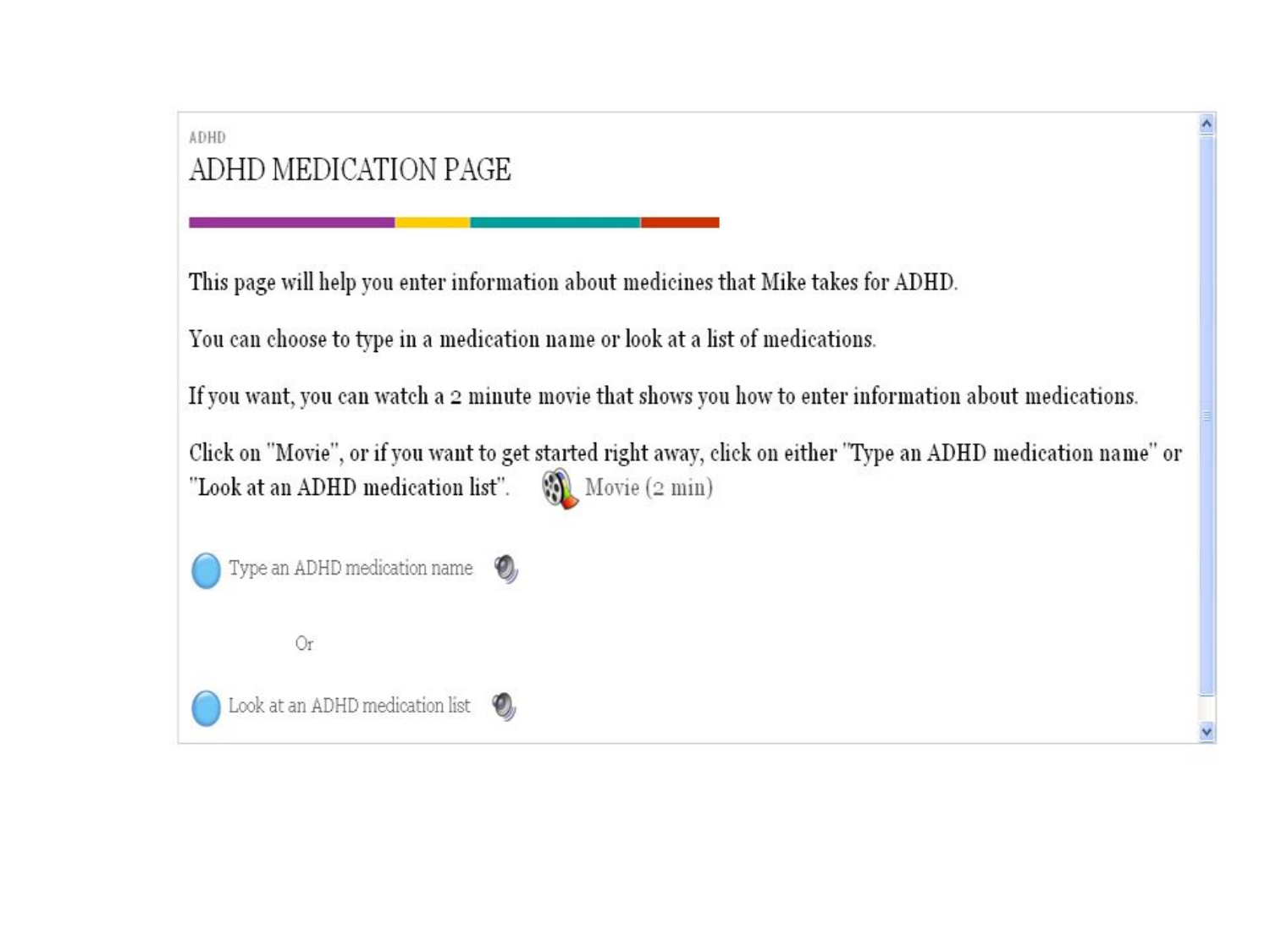

## Slide 5
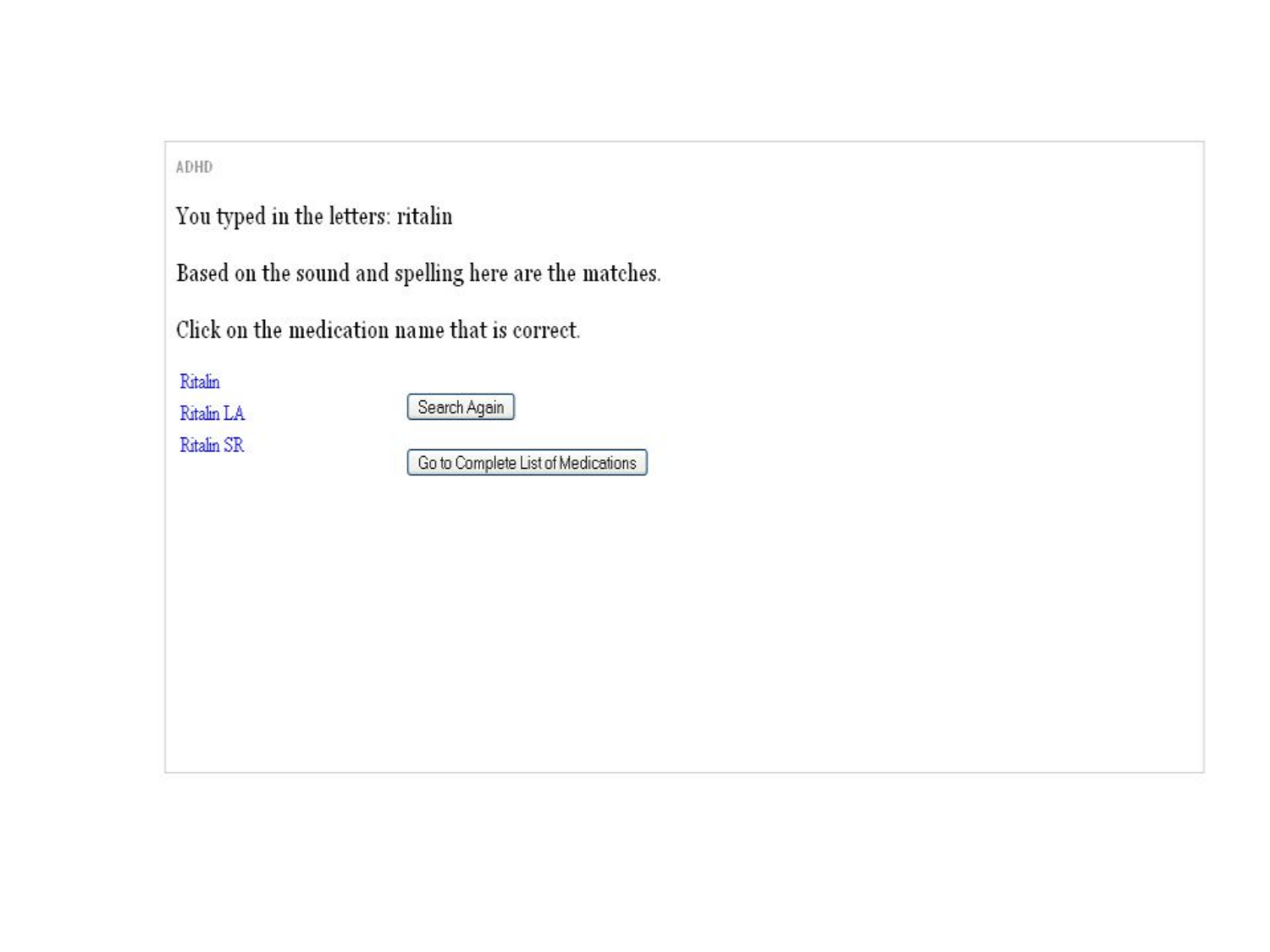

## Slide 6
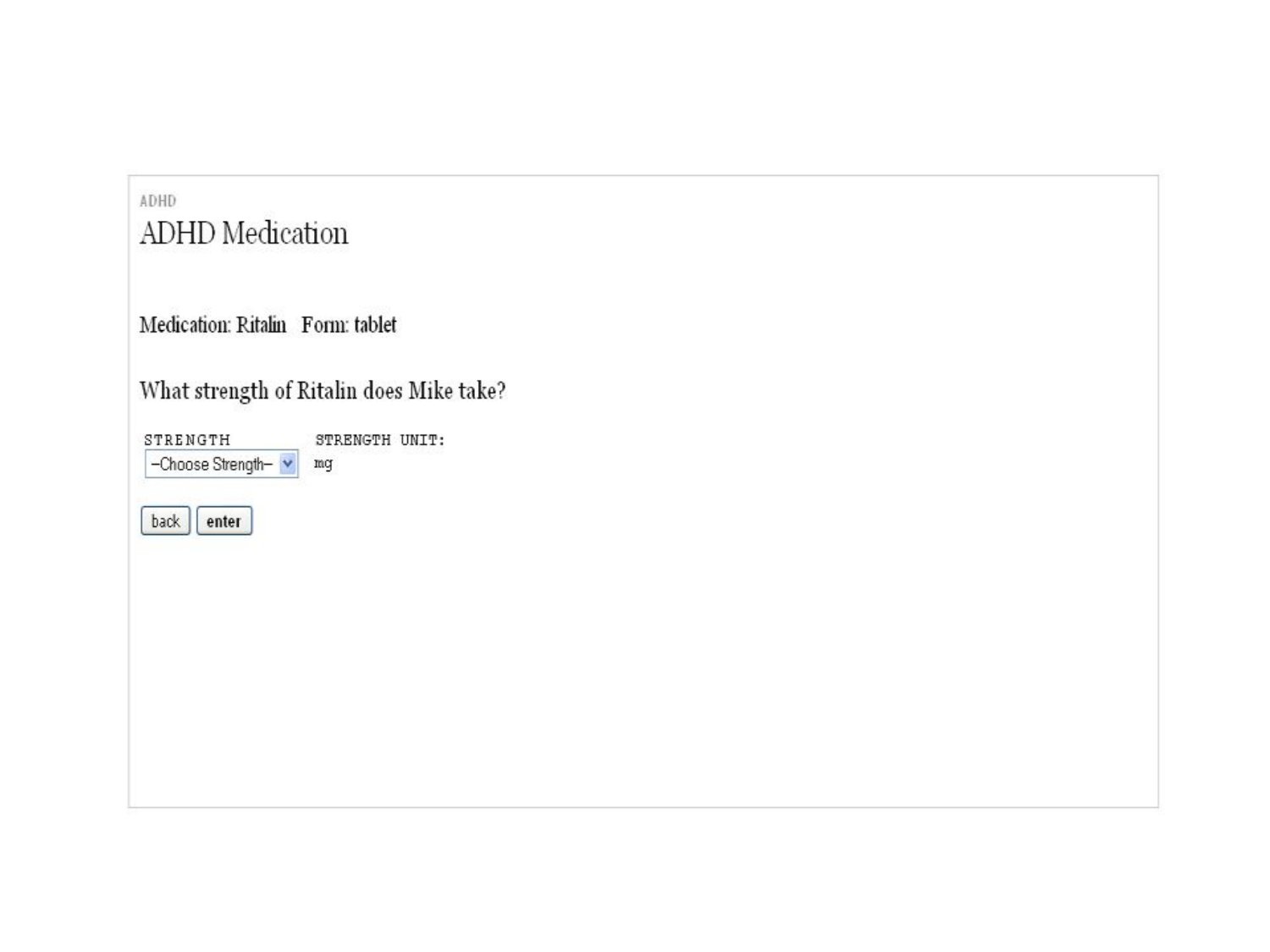

## Slide 7
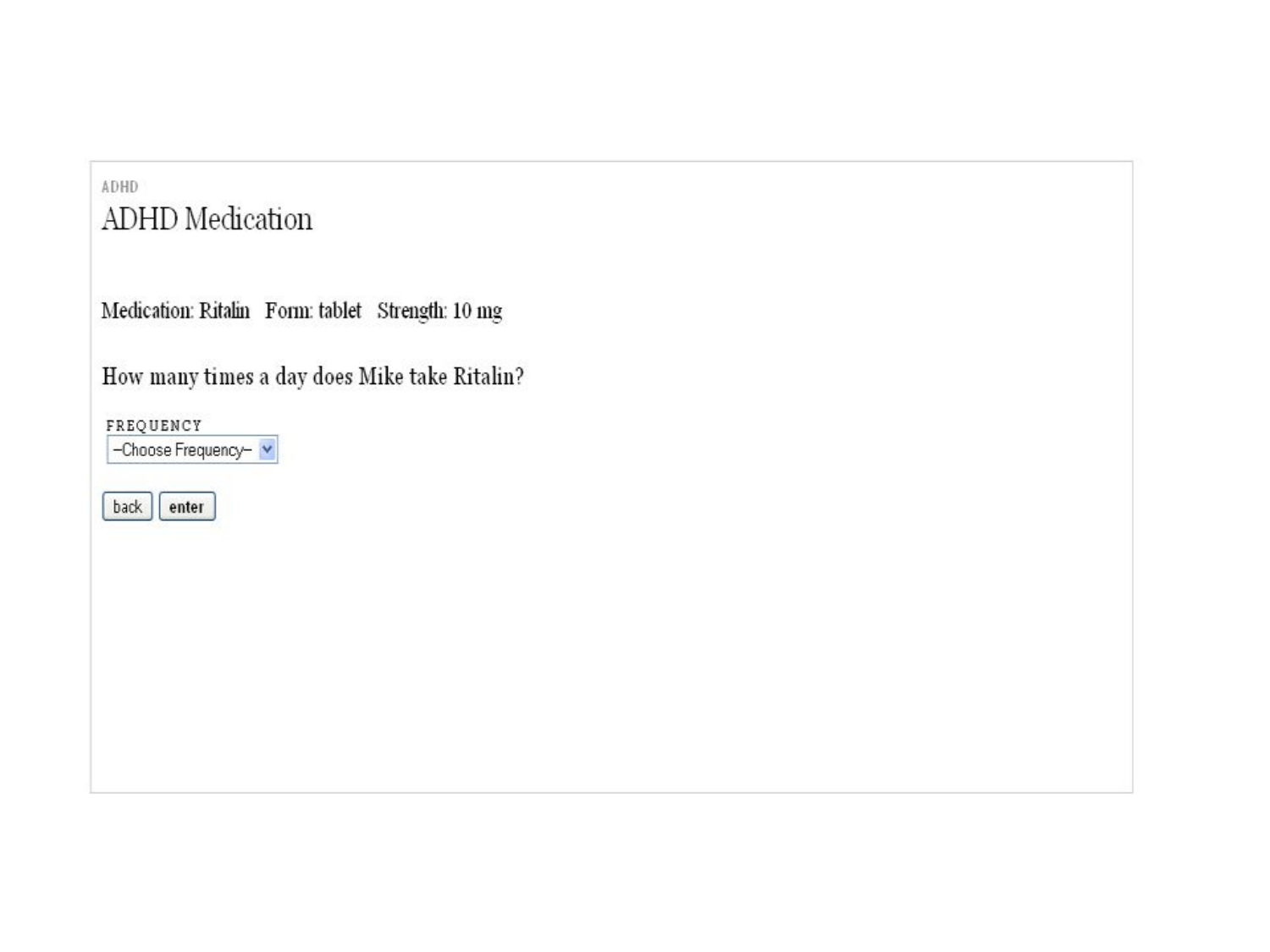

## Slide 8
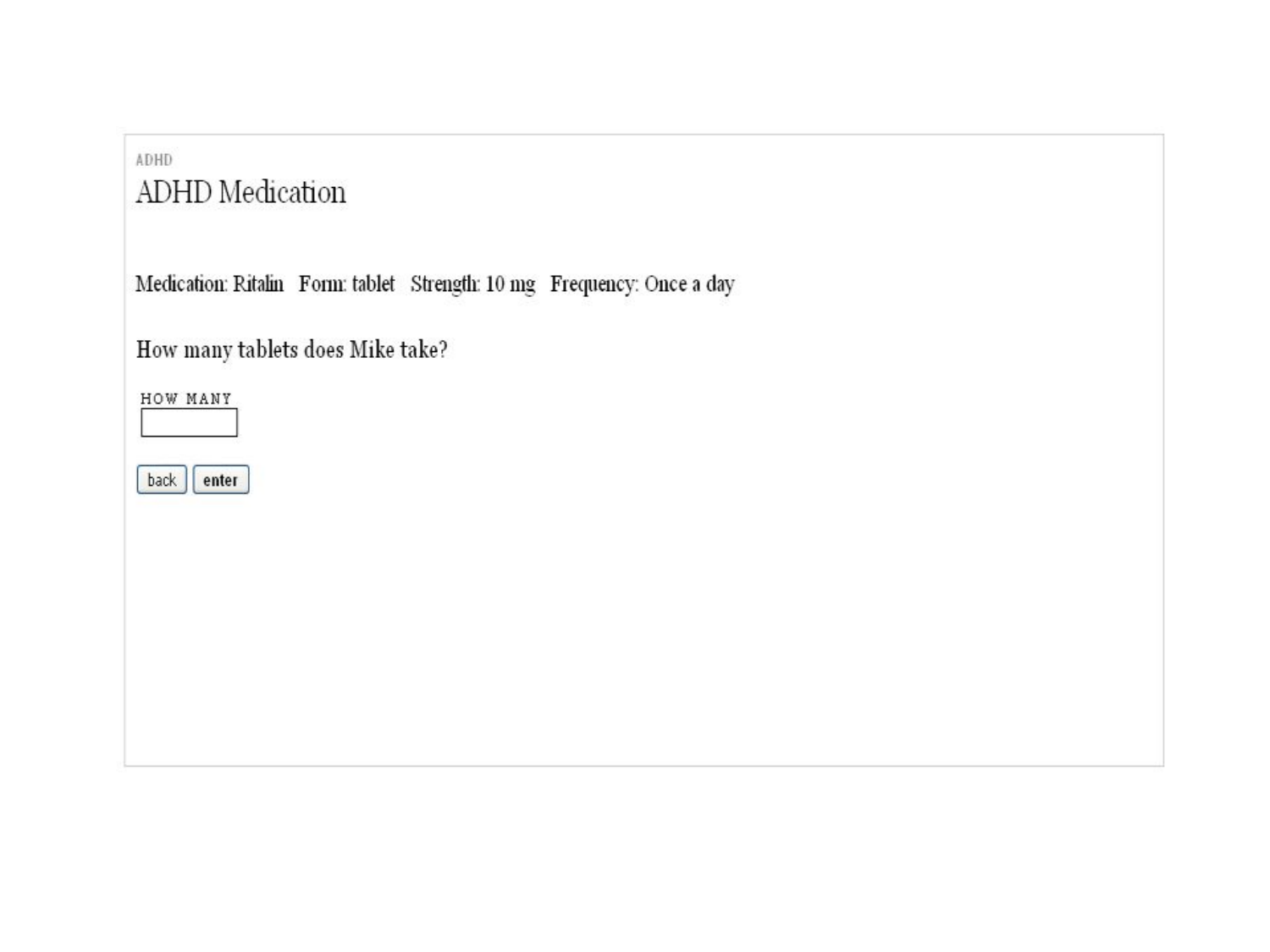

## Slide 9
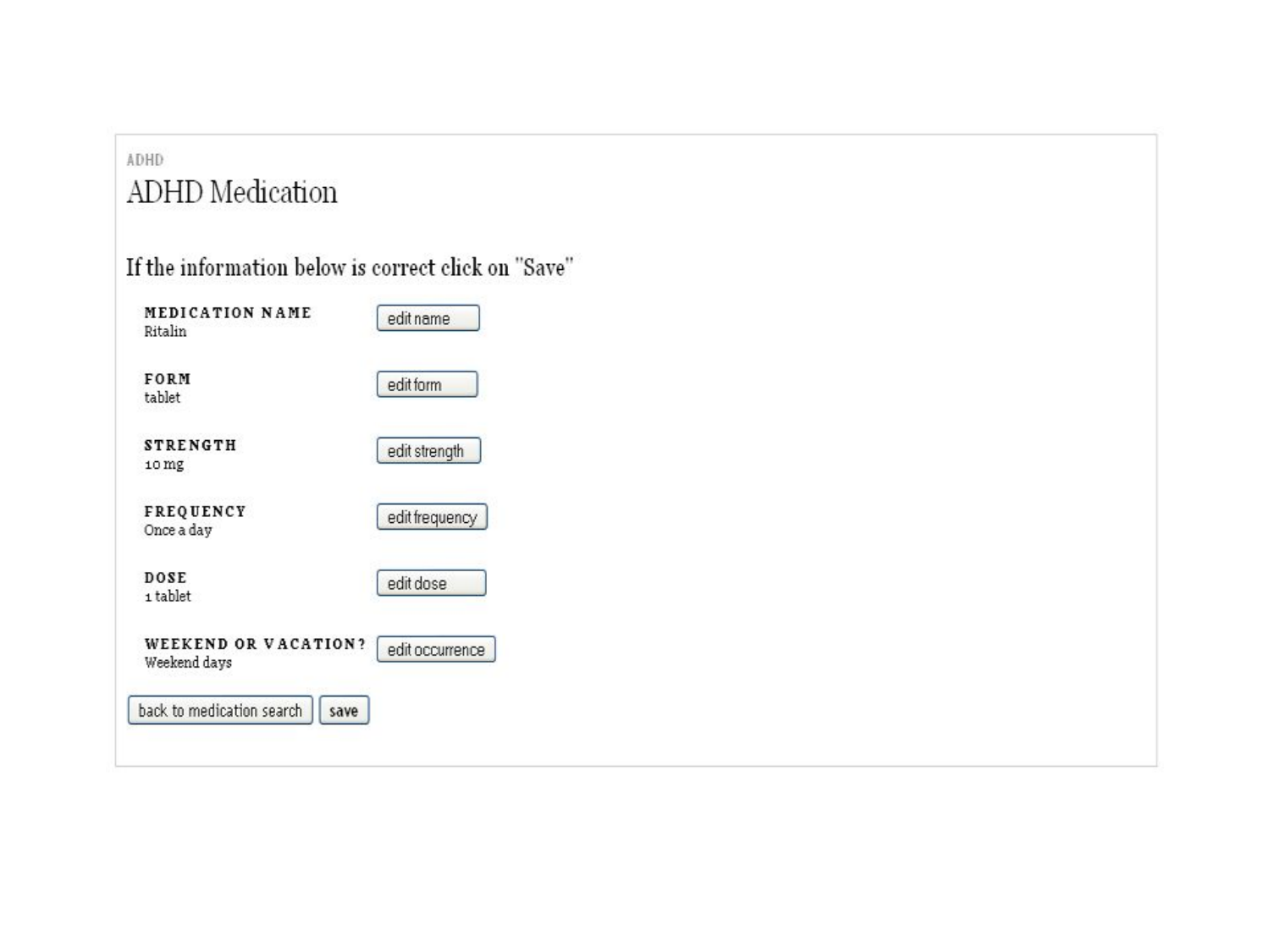

## Slide 10
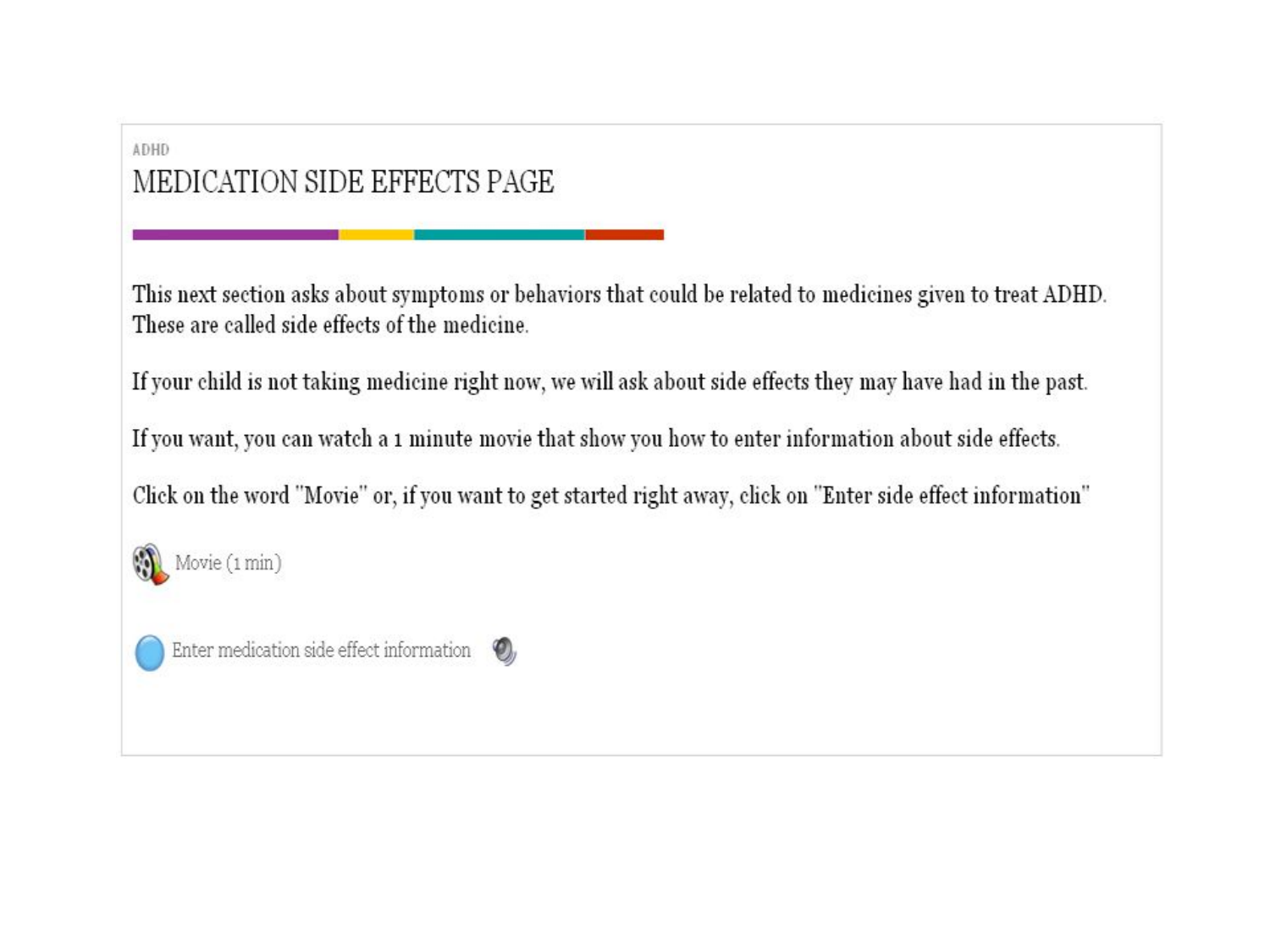

## Slide 11
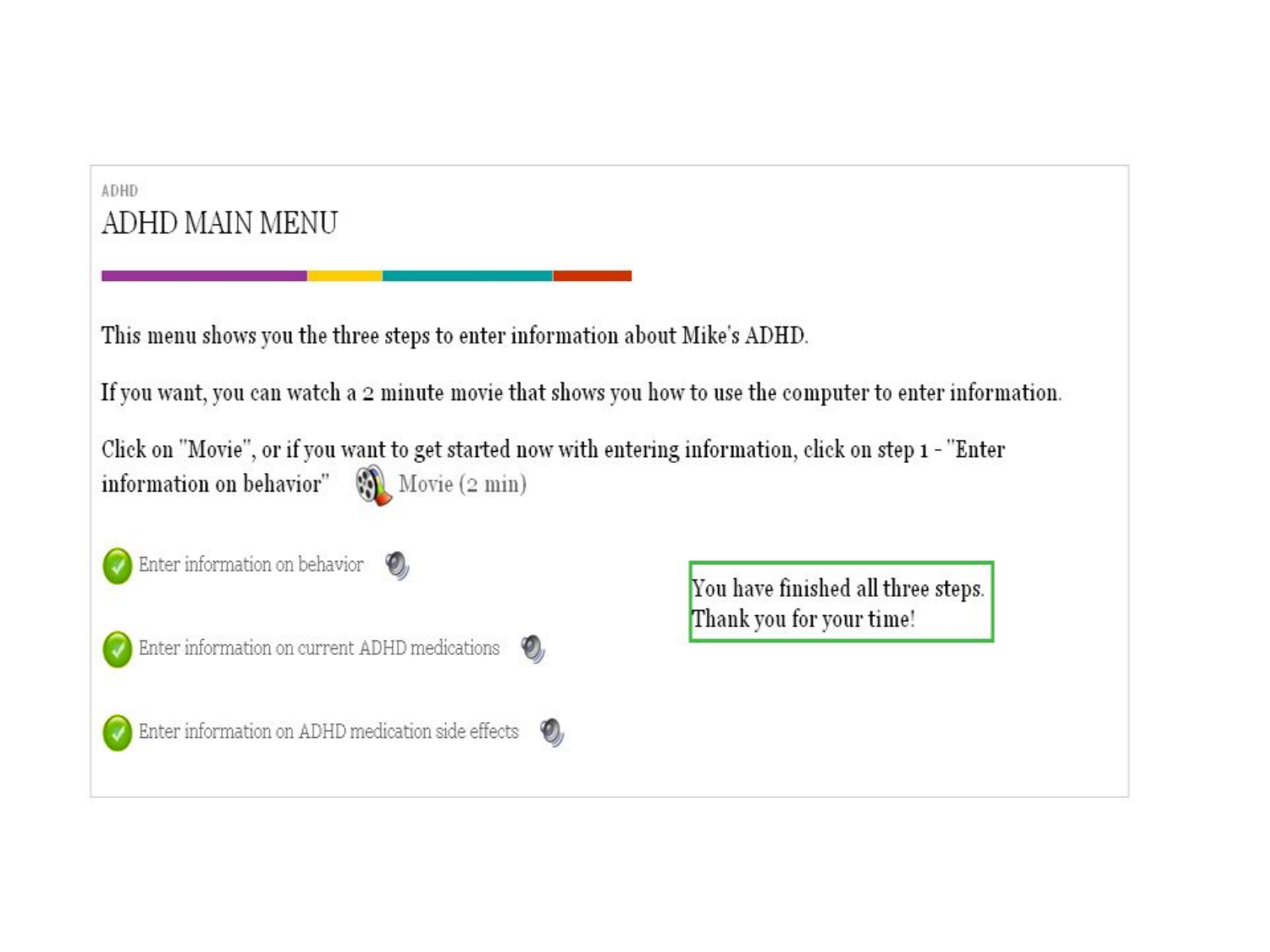

Supplement: Supplementary file 3 [file jmir_v12i3e36_app3.ppt]
